# Experiences of health care in women with Peripartum Cardiomyopathy in Sweden: a qualitative interview study

**DOI:** 10.1186/s12884-016-1178-3

**Published:** 2016-12-08

**Authors:** Harshida Patel, Maria Schaufelberger, Cecily Begley, Marie Berg

**Affiliations:** 1 Institute of Health and Care Sciences, Sahlgrenska Academy, University of Gothenburg, Gothenburg, Sweden; 2 Department of Molecular and Clinical Medicine, Institute of Medicine, Sahlgrenska Academy, University of Gothenburg, Gothenburg, Sweden; 3 School of Nursing and Midwifery, Trinity College Dublin, Dublin 2, Ireland; 4 Centre for Person-Centred Care (GPCC), University of Gothenburg, Gothenburg, Sweden

**Keywords:** Peripartum cardiomyopathy, Pregnancy, Heart disease, Childbirth, Qualitative, Women’s experiences

## Abstract

**Background:**

Peripartum cardiomyopathy is often associated with severe heart failure occurring towards the end of pregnancy or in the months following birth with debilitating, exhausting and frightening symptoms requiring person-centered care. The aim of this study was to explore women’s experiences of health care while being diagnosed with peripartum cardiomyopathy.

**Method:**

Qualitative interviews were conducted with 19 women with peripartum cardiomyopathy in Sweden, following consent. Data were analysed using qualitative content analysis. Confirmability was ensured by peer-debriefing, and an audit trail was kept to establish the credibility of the study.

**Results:**

The main theme in the experience of health care was, ‘Exacerbated Suffering’, expressed in three subthemes; ‘not being cared about’, ‘not being cared for’ and ‘not feeling secure.’ The suffering was present in relation to the illness with failing health symptoms, but most of all in relation to not being taken seriously and adequately cared for by healthcare professionals. Women felt they were on an assembly line in midwives’ routine work where knowledge about peripartum cardiomyopathy was lacking and they showed distrust and dissatisfaction with care related to negligence and indifference experienced from healthcare professionals. Feelings of being alone and lost were prominent and related to a sense of insecurity, distress and uneasiness.

**Conclusions:**

This study shows a knowledge gap of peripartum cardiomyopathy in maternity care personnel. This is alarming as the deprecation of symptoms and missed diagnosis of peripartum cardiomyopathy can lead to life-threatening consequences. To prompt timely diagnosis and avoid unnecessary suffering it is important to listen seriously to, and respect, women’s narratives and act on expressions of symptoms of peripartum cardiomyopathy, even those overlapping normal pregnancy symptoms.

## Background

In most women, moving through pregnancy, labor, birth and the postpartum period is a process associated with health and happiness, but some can be affected with illness. Peripartum cardiomyopathy (PPCM) is one such condition, associated with severe heart failure (HF), that can be life threatening. Defined by the Heart Failure Association of the European Society of Cardiology Working Group, PPCM is*: An idiopathic cardiomyopathy presenting with HF secondary to left ventricle systolic dysfunction towards the end of pregnancy or in the months following delivery, where no other cause of HF is found. It is a diagnosis of exclusion. The left ventricle may not be dilated but the ejection fraction is nearly always reduced below 45%* [1].

The incidence and prognosis of PPCM varies globally [2]. The true incidence is unknown, as the clinical presentation varies. Current estimates range between 1:299 (Haiti), 1:1000 (South Africa), and 1:2500- 4000 births (USA) [1–4]. Data on the prevalence of the disease in Europe are scarce [5]. Assuming an incidence of 1 : 3500 to 1 : 1400 births would yield an expected incidence of up to 300 patients per year in Germany, with severe, critical cardiac failure in around 30 [6]. The incidence in Sweden has been estimated to be 1:9191 births [7].

Major symptoms of PPCM are those of HF and include fatigue, shortness of breath, and fluid retention. Diagnosis is often missed or delayed in pregnant women as symptoms are similar to those of hemodynamic changes in normal pregnancy or early postpartum period [1, 8–11].

Experiencing PPCM causes suffering and can, as in most severe illnesses, subsume all of the patient’s energy [12]. To relieve suffering due to illness is one of the main responsibilities of healthcare professionals [12]. Health care laws and guidelines in Sweden [13, 14] stress the importance of taking into account the patient’s knowledge, experiences and desires and discerning the individual’s unique needs for care and support. Alleviating suffering in relation to pregnancy and childbirth requires healthcare professionals to listen actively to, and base care upon, the patient/client’s narrative [15, 16]. Some outdated guidelines exist for the care of women with PPCM that include recommendations on the need for a good nursing relationship, correct diagnosis, and the provision of information and support [17]. Previous studies describe clinical and treatment issues in the areas of antenatal care, childbirth and postnatal care, but no studies have, to our knowledge, drawn attention to the experiences of received care in women with PPCM, apart from two qualitative studies [18, 19] that have analyzed patient internet postings.

Understanding “what it is like” from the sufferer’s perspective has been acknowledged as a foundational starting point for caring [20]. It is important, not only to have objective knowledge about a patient’s condition, but also to understand the subjective experience and meaning of illness from the affected person’s perspective [20]. The need for more research to acquire knowledge from those affected by PPCM is therefore essential. Recently, we published the first part of our study presenting women’s experiences of symptoms of PPCM [11]. In order to give optimal care, more knowledge from the insider perspective is crucial [21]. The aim of this study was, therefore, to describe and explore women’s experiences of received healthcare while being diagnosed with PPCM.

## Methods

In order to understand the “insider” perspective [21] from women with PPCM a qualitative method was used.

### Setting

Maternity care in Sweden is free for all women. The majority of births are in hospital, with approximately 0.1% occurring at home [22]. Care in normal physiological pregnancy and childbirth is carried out by midwives. In case of complication women are referred to a gynecologist/obstetrician with continued involvement of midwives in the care.

### Participants

Medical records from Western Sweden were used to select a purposive sample of 25 women [23], who had had a diagnosis of PPCM, based on the criteria from the European Society of Cardiology [1], between 2005 and 2012. The first author (HP), who had no professional caring role with the participants, contacted them by telephone, asked if they were willing to participate in the study and checked that they were able to speak Swedish. Two were excluded because the criteria for PPCM diagnosis were not fulfilled; one woman had difficulty with the Swedish language; three declined to participate due to lack of time.

### Data collection

Data were collected through unstructured interviews, which took place a median of 43 months after the index pregnancy (range: three months to seven years). Characteristics and childbirth outcomes for the 19 interviewees have been previously published [11]. HP conducted all interviews either in a private room outside the outpatient clinic (*n* = 13), or as telephone interviews (*n* = 6), when it was difficult for the women to give time for a face-to-face meeting. A trusting relationship with each woman was established by listening carefully to, and accepting, her narrative. All participants were asked to complete a short series of demographic questions, and then open questions were asked in order to capture in-depth experiences: ‘Will you describe your experiences of the healthcare?’ ‘Probes’ were then used such as: ‘What do you mean?’ or ‘Can you elaborate further?’. Interviews took 20 to 90 min and were recorded and transcribed verbatim. Any noteworthy non-verbal cues such as crying or laughing were documented during the interview.

### Data analysis

Transcripts were compared with the audiotapes to ensure accuracy, and relevant information from field notes and memos, such as non-verbal cues or reactions, were noted. Inductive content analysis [24, 25] was used to analyze all interview transcripts and emerging categories were combined into themes, through more in-depth thematic analysis. The inductive stance in the analysis of content in the data guided us to be open minded, conscious about our preunderstandings, and producing findings as close as possible to the data given, in line with our constructivist standpoint. All data analysis steps were performed individually by HP and MB and then discussed to ensure agreement of the description and interpretation of data. Initially, interview texts were read to get an overall ‘feel’ for the data. Then, the text was read again and sections relating to the aim of the study were identified independently. Units of analysis that described experiences of care were then separated, and notes were written in the margins, describing aspects that related to the women’s experiences of care. In the next step units of analysis were transferred to separate sheets with the written notes, and headings summarizing all descriptions of women’s experiences of care were inserted, by HP, MB and CB. All quotations used to illustrate women’s experiences were then translated into English. To ensure reliability, all researchers discussed during all stages of the analytical process the various interpretations. The stages and processes of analysis were documented precisely in an audit trail, to make it possible for others to follow the process. Confirmability was measured by external checks (“peer-debriefing”) conducted by colleagues who were familiar with qualitative research. Finally the analysis ended up in identification of three sub themes forming one main theme.

## Results

The analysis of women’s descriptions revealed three subthemes; ‘not being cared about’, ‘not being cared for’ and ‘not feeling secure’ which all together formed the overall meaning covered in the main theme: ‘Exacerbated Suffering’. Negative care experiences dominated often before the PPCM diagnosis was confirmed. Some positive care experiences were described and are presented in respective subthemes. In the following, exemplifications and description of the three subthemes is given including quotes illustrating women’s own words to ensure credibility, identified by the use of code numbers: P1, P2 etc. Finally, the main theme is described.

### Not being cared about

‘Not being cared about’ encompasses not being taken seriously, which includes not being seen or listened to, or to be ignored and misjudged. Frustration in relation to such experiences all together contribute to a common feeling among the women; that health professionals neither believed nor understood the problem; this could be either at ante-, intra- or postpartum care.

Repeated disappointments over perceived nonchalant encounters with maternal healthcare staff, especially midwives, were described. This comprised neglect due to explaining the symptoms of PPCM as normal pregnancy symptoms. For example: chest-congestion and breathlessness was explained as the humidity in summertime compounded by a baby pressing on the lungs (P7), or as stress and anxiety (P9); breathlessness after the birth was interpreted as constipation (P5), and was attributed to recent childbirth (P16); and resting heart rate at 90 beats/min during pregnancy was explained as normal in spite of causing distress, and abnormal weight gain and enormous fatigue had no explanation (P13).

Not being listened to in spite of complaints of ill health generated a vicious cycle of suffering, demonstrated by the following quotations: *No one believed me… I could not enjoy the pregnancy because I was in stress and worried all the time… difficult to make myself heard…*(P1). //:”…*things started getting crazy*… *my feet and ankles and calves became so swollen…Again I went to the midwife, but she told me it was “normal”* (P4).

Feeling humiliated, annoyed and misjudged was obvious in relation to the inability to receive serious attention. For example, one woman explained how she, while seeking emergency care for symptoms was sent home by the physician with symptoms attributed to normal pregnancy. The suggested solution was to be more physically active and exercise to manage the symptoms. Another woman stated:

*“I was not feeling well throughout my pregnancy…heart palpitations, but midwife said it’s OK … it’s common…I was exhausted but they said it is due to the pregnancy. I had panic attacks, I went in to the ED… the ECG was pathological, but was sent to the psych unit and then home” (P19).*

Such an apparent lack of response to complaints caused the women to blame, partly, the healthcare professionals. This was shown in expressions of broken trust during caring encounters: “*All these happened because they did not listen to me… They attributed my symptoms as mental and emotional*” (P5). It also became evident when the health care professionals relied on results of objective tests more than women’s subjective experiences and complaints. This appeared in a woman who felt that the midwife had a blind faith in the guidelines. “…*I was in the upper limit but I normally have low blood pressure, so the difference was greater for me but it was normal for her (midwife)… hypertension went unnoticed, which might have resulted in PPCM*” (P15).

Women described how their symptoms were dismissed, which gave them a feeling of rejection and of not being cared about: “*I asked for the note on sick leave as I was extremely tired, but she (gynecologist) did not respect my wish…”* (P5). One woman felt ‘like a pile of garbage’ without any hope for life and the future (P14). Additional suffering occurred when midwives appeared to hide behind the routine and care culture; this is obvious in the following quote. “*I told them about my shortness of breath…I was sent home…I was suffering…3 days later they discovered my heart failure” (P7).*

Women who felt that they were not taken seriously and did not receive adequate responses from healthcare professionals, in their complaints described how they had to fight to get proper care. This is exemplified in the following quote:

*“A doctor who listened to my lungs, he said it was ok… I felt overly exhausted, but they (healthcare professionals) kept telling me that I had just been through childbirth and all this was normal… I know today that if they’d listened to me, I might not have been affected by this (PPCM)”….* “*Later on, at the ICCU it’s the same feeling as earlier in the maternity ward. I asked her (nurse) for a diuretic so I can pee out because I was terrified that I would get pulmonary edema again. She (nurse) replied that it is not necessary. Well, I felt that it was crackling in the lungs but she insisted, but finally…I received a diuretic and peed 2 liters” (P18).*

Some women felt that the midwives ignored their symptoms, which led to deterioration of the condition and to prolonged hospitalization. One woman described how the postnatal ward staff only observed the child and had a focus on breastfeeding but really did not keep a track of her condition. Another woman described her feelings when her pain and suffering was ignored: *“I had to nag and fuss for pain medication… it hurt to breathe, but they said ‘you just want painkillers and to avoid breastfeeding.’ I was very sad and felt terribly bad, felt like a drug addict (P17).*”

An example of ‘not being cared about’ comprising a feeling of not being taken seriously was apparent when one obstetrician was reported to have misjudged a woman’s symptoms of ‘popping and crackling in the lungs, and squeezing chest.’ The decision was to wait and see the further development instead of initiating investigation. The woman stated: *“… Deep down in my heart I knew I was dying, though they do not see me if I die now, difficult to get them to listen. I was looking at the door waiting for someone to come and see how bad I was” (*P12).

Some positive care experiences were identified in the narratives. Women who believed they had received adequate help quickly enough, and were taken seriously, expressed their gratitude and credited healthcare professionals for being responsive, attentive and saving their lives. It was also obvious that the majority of women expressed their satisfaction with care, once they received care by cardiologists who confirmed their symptoms were related to PPCM.

### Not being cared for

The theme: “not being cared for” comprises not having been given needed physical or emotional assistance and lack of adequate information. One of the examples can be read as:

“*After the ECHO cardiography, I walked by myself through the long tunnel, although he (technician) saw how bad I was…I had no idea, but he (technician) just said, don’t you know, that you have heart failure” (P9).*

‘Not being cared for’ also seemed to take the form of a power struggle, disguised by routine and by lack of knowledge about PPCM in midwives. As shown above, the overlapping symptoms of PPCM to normal pregnancy were explained as normal by midwives in spite of women’s suspicion of something serious going on, and then showed up later on as a serious health problem. Women experienced anxiety and needed an adequate explanation and emotional assistance as comfort from midwives.

The patterns of ‘not being cared for’ differed somewhat between women having given birth before and those encountering childbirth for the first time. Multiparous women could compare their symptoms with an earlier pregnancy, but primiparas, such as the following quotation shows, could not differentiate symptoms of the normal pregnancy and the deviations due to PPCM:

*“… I suspected nothing because no one said anything… one would think it is normal while carrying twins. After the birth I gained even more water and looked like colossus… they said it is common… I could not walk in the corridor, when I was discharged. I said, excuse me, but should I breathe like this? I had to go home anyway.”* (P6).

Another example of not being cared for is perceived lack of information, or insufficient information. Repeatedly in the women’s narratives healthcare professionals were described as being perfunctory when they did not provide an adequate explanation. This is obvious in the following quotations of women who sought acute care and met physicians who, after seeing their symptoms, performed an investigation and sent them home in spite of ongoing feelings of malaise:

“*… ‘ECG looks strange, but you have a small child so it is best that you go home.’ The next day the error was discovered and I received a call, they had missed that I had fluid in the lungs…it was horrible” (P15). //… “…they said it is not so strange (breathing difficulties) because I had lost blood”* (P17).

Perceived deficiencies were also revealed in coordination and assistance once they sought care. This was expressed as:

*“I was at home less than 24 hours after discharge from the hospital after childbirth. I could not lie down so I sat up all night…I went to the emergency room with 4 days old babies. After ECG we had to go to another hospital (-), because they had no maternity ward here (-). No one knew what was wrong with me. After my arrival at gynecology-ward, I felt, I was not welcome there…I was left alone” (P17).*

Some women, despite their need for rest, were placed in a room among healthy mothers with screaming babies because of shortage of beds, which was experienced as demonstrating poor care due to organisational failures. ‘Not being cared for’ was also seen as the struggle these women had to cope with in everyday life despite their ill health, with additional suffering caused by the perceived lack of understanding by healthcare professionals; *“I was referred to a special clinic… I had to go there every day for check-ups after leaving two other kids in kindergarten and it was very strenuous” (P9).* Other women expressed as quoted:

*“I wanted to stay home and rest for a day but …they (neonatal nurses) said that my daughter needs her parents, specially a mum. I don’t know if they noticed but I was sitting there and was feeling very uncomfortable. I told them (nurses) that I had been in the intensive care unit after the childbirth… I had HF and I needed to be with my elder child and just needed some rest… but they did not understand” (P18).*

Some positive and satisfactory experiences about being cared for were identified in the narratives. These were experienced mainly at the cardiac care unit after a confirmed diagnosis of PPCM that explained women’s symptoms and led to the correct treatment and rapid retraction.

### Not feeling secure

The third sub theme, ‘not feeling secure’ covers women’s feelings of insecurity in relation to their own health and well-being and their perceptions of how they were treated, either not being cared about or not being cared for, which resulted in distrust of healthcare professionals. For most women, PPCM remained unidentified at an earlier stage of pregnancy despite typical symptoms, and was discovered first after childbirth. These women associated such late diagnosis with incompetence in the healthcare professionals, and were upset that, despite persisting symptoms, no one tried to examine their story further.

For two of the women their symptoms had been interpreted to be a mental disorder. They were referred to the psychiatric unit and described the mixed negative feelings such as rejection, violation, insecurity and distrust relating to the health care professionals’ knowledge and attitude. One of the women who had gone through in vitro fertilization to become pregnant expressed her feelings of regret as a consequence of insecure and distrusting care: “…*Is it worth expecting a baby after all the trouble with IVF and now this…?”*(P8).

The following quotations from two women express how the feeling of insecurity was strengthened by not getting appropriate care and not being understood with one’s subjective experiences: “*If I die, no one will find me .*.. (P18). *“ASCH! she (X-ray nurse) said… you won’t suffocate… I was afraid to die… She forced me to lie down … she said ‘we can see if you stop breathing’ (P12).*”

Negative encounters with midwives mediated a sense of insecurity that led to the avoidance of seeking help even when serious problems like oliguria occurred. This is exemplified in the following narratives:

“*…feeling a little bit hesitant to contact, and explaining myself all the time was hard …it’s unpleasant… she (midwife) did not respond adequately” (P18).* //: *“If they had taken a blood sample, they might have discovered that my heart was affected. I stopped peeing but I didn’t call the nurse-midwife because I had already called so many times and received an answer that there was no reason for worry. There was nothing else I could do but believe in midwives and just wait” (P12).*

Some women, who were sent home following consultation in specialist clinics, needed to seek emergency care only a few hours later because of residual symptoms. These women experienced insecurity relating to disappointment towards health care professionals, as expressed in the following quote:

“…*I started coughing at night…sought care at the primary care…referred for further investigation…had to wait a whole day in spite of breathing difficulties…If he had …given me diuretic earlier… I am totally convinced that they could have stopped the deterioration.” (P15).*

The women needed repeated information related to their severe condition both in pregnancy and after birth when being occupied with a new born baby made it more difficult to capture fully the meaning of getting this serious condition. A feeling of insecurity was also connected to women’s perceived lack of timely and true response to them about what was going on and what was going to happen.

Some contrary findings were seen, where women indicated a feeling of being secure. This was experienced by women who had received adequate help quickly enough by healthcare professionals they believed to be competent. This is shown in the following quotations: “*I felt like the world’s elite team was there for me during labor*” (P7). Feelings of security were also related to simple things, such as having a contact number to the cardiac nurse at the nurse-led outpatient clinic, cardiologist or knowing that they can call any time to the cardiac ward for advice. Additionally, although women were satisfied with the follow-ups by specialist nurses, they desired more follow-up check-ups by the cardiologist. All together, these positive feelings seemed to give the women hope and strength to fight on and look forward to a brighter future.

### Exacerbated suffering

A continuous thread through the three subthemes: ‘not being cared about’, ‘not being cared for’ and ‘not feeling secure’ is identified, which is an ‘exacerbated suffering’. This suffering was added to the already existing suffering related to the symptoms of PPCM and was caused by several uncaring actions from healthcare professionals. It comprised neglect of symptoms, avoidance of the women in terms of not seeing, listening to or respecting the women’s complaints. It also comprised lack of practical assistance, and it meant that the women had to seek other care, such as emergency care. Although not purposefully, the health care professionals caused and exacerbated women’s suffering by, in the women’s view, neglecting and normalizing the emerging symptoms of PPCM. This in turn can be explained by the fact that no proper care guidelines on routines of identifying and treating women with PPCM existed.

## Discussion

This study aimed to reveal experiences of care in relation to symptoms and diagnosis in women with PPCM. Although the negative care experiences dominated in women, some positive experiences were also described. The findings showed that women’s suffering was not only caused by symptoms of PPCM but also related to the care received. The main theme, *exacerbated suffering,* emerged from three subthemes: *not being cared about, not being cared for* and *not feeling secure*. The exacerbated suffering caused from the standpoint of women; misinterpretation of symptoms leading to delayed diagnosis led to a complex situation with expectations, trust and paradigm clashes between women and their attending midwives and physicians. Women suffered from the lack of care and the denied affirmation of suffering.

The results show that the focus of healthcare professionals is from a predominantly biomedical, and not holistic, perspective. It is difficult to say how much of the midwives’ and obstetricians’ responses are affected by the rarity of the condition and how much depends on individual competence, personality or organization (staff shortages, stress, etc.). It is understandable that perfect care is not given on occasion, due to shortage of staff, perhaps, but it is not justifiable. It appeared, however, that the encounters with healthcare professionals were generally improved after the diagnosis was established. Positive experiences at cardiac unit could be explained by the staffing level and competent personnel in cardiac disease.

Although some women had positive experiences from maternity care in the current study, they reported an over-focus on ‘whitewashing’ of their symptoms/complaints. This confirms similar findings from earlier studies [19, 26] and may also serve as an explanation for the negative feelings women expressed. Psychological stress related to feelings of not being cared about might have resulted in the exacerbation of PPCM causing pulmonary edema in some women, which has been shown in previous research [27].

Dissatisfaction with care in women during pregnancy and postpartum has been described in literature, related to suffering from many physical symptoms, little involvement in decision-making and lack of support by midwives [28]. The main reason for frustration in women with PPCM has been shown to be fear, anger with the nursing staff for being ignored, dismissed, and neglected, and misdiagnosis [29]. A qualitative study of seven adults with Long QT Syndrome also found that they suffered from misdiagnosis and ‘not being believed’ [30], which led to feelings of uncertainty and dissatisfaction with their medical care. The majority of women in our study described similar experiences of failing support from midwives, stressing the importance of being attentive to the women’s narratives.

Our previous research in women with PPCM showed that symptoms overlap with normal discomforts of pregnancy, and thus create space for clinicians to overlook the seriousness of their situation [11]. Early measurement of NT Pro-BNP marker in women who present with worsening dyspnea during pregnancy or the postpartum period should be encouraged for early suspicion and diagnosis of PPCM [31]. The increased knowledge in healthcare professionals is essential to influence and facilitate positive caring.

Ekman et al. stress the importance of person-centered care (PCC) to increase patient satisfaction [32], which is based on the narratives of the sufferers. The midwives have general knowledge of how a pregnancy manifests itself, but it is only a ‘person’ who constantly lives with signs of ill-health that has a more nuanced picture of their symptoms and can describe a full picture of it from different dimensions [11, 32]. A philosophy of care that is consistent with PCC, describes how meetings with patients should be focused on the patient, who is an expert on herself [33]. Becoming a parent is enough challenge to one’s identity, and emotions such as anxiety, uncertainty and fear are common [34]. The additional challenge of coping with suffering from a severe condition such as PPCM increases the feeling of life’s uncertainties. Moreover, additional experiences of not being cared for in adequate manner leads to unnecessary suffering in the women. Health professionals should consider every woman’s experiences as unique and address their feelings with respect.

Previous research has found that women at high risk are more vulnerable during pregnancy and/or childbirth [35]. A positive encounter between the women and healthcare professionals can increase their self-confidence, reduce suffering and restore dignity [34]. The philosopher Levinas’s [36] work has explained and justified why to be there for the other (in our study the woman) is health professionals’ indisputable duty. According to Levinas, it is by bringing together the dimensions of care and the components of the person that the concept of ‘to take care of the other’ finds its whole significance and consistency. In our study, knowledge gap, unconscious behavior, non-reflective approach or judgmental attitude might have lain behind the causes of suffering and the feelings of hopelessness and despair expressed in women’s narratives. This type of violation causes ill health, a reduced sense of self-worth and lack of trust [37] resulting in avoidance of seeking care even in case of emergency. This finding demonstrates clearly that the health professionals did not always use PCC communication approaches, but rather made their own interpretation of women’s symptoms. Inappropriate treatment may give rise to negative thoughts for future pregnancies. Similar to experiences of women in our study, negative encounters with healthcare professionals are described as being brushed off, dismissed, and ignored by other researchers [29]. Researchers from this study found that women with PPCM were told that they had anxiety, acid reflux, bronchitis, pulmonary thrombosis or were simply experiencing pregnancy. In contrast, a study of women who had a prior diagnosis of congenital heart disease showed that these women had a very strong faith in their clinicians and their abilities, perhaps because the diagnosis was known many years before conception [38]. As PPCM that is unknown before pregnancy can affect and shatter the existence of a woman in multiple dimensions, the notion of care must take into account appropriate ‘interventions’ that will meet the needs of every single person. Only through a good dialogue can midwives take part in the women’s story and enter into the women’s problems and world, to meet their needs and alleviate suffering.

## Strengths and limitations

A main strength of this study is its originality. It is, to our knowledge, the first study presenting experiences of care in women with PPCM. The greatest utility of this study lies in the transcendence of knowledge between two expertise areas, i.e. cardiology and midwifery that brings new insights to midwives. This extended view can lead to better caring and treatment as well as more skillful diagnoses. The use of women’s narratives is an important scientific tool in order to understand the meaning of developing PPCM. Although the sample consists of just 19 women, and is collected from a single setting, we believe that our data are quite representative of women with PPCM in Sweden, given that it is a rare disorder. Most women delineated similar experiences of care, in spite of the time after diagnosis varying between 3 months and 7 years after received diagnosis of PPCM (thus confirming data saturation). The narratives on experiences of care in this study are retrospective; women had to look back and remember, and while doing so new meanings can be discovered and added to earlier ones. Although women’s experience of care cannot be questioned, threats to the validity of the narratives cannot be excluded, e.g. risk of recall bias. Some women might not remember the whole picture of negative and/or positive experiences related to care in PPCM. However, we could not find any difference in narratives based on time since diagnosis, but it is possible that women may have had selective memory and remembered what they wanted to remember, either consciously or unconsciously. It is difficult to make definite conclusions because women with good encounters with healthcare professionals mentioned about care in general and, looking back at the hospital stay, gave specific examples of situations with good care. Women with bad encounters talked only about individual examples and did not mention how they perceived care in general, despite verbal prompts to do so. However, regarding recall bias, previous studies found that long-term maternal recall is both reproducible and accurate in relation to pregnancy and delivery [39, 40].

A study limitation is that the study only included Swedish-speaking women. Of course more studies need to be done involving women who do not understand and speak Swedish, to provide good care for all women regardless of origin.

## Conclusions

The findings from this study broaden our understanding of the hardship and personal suffering in women experiencing PPCM, and shows that a delayed diagnosis of PPCM may have great impact on women’s daily life and health. Knowledge of the medical signs and implications of PPCM may contribute to good care but it is not a necessity. Instead, we highlight the importance of the basic values and the need for a person-centered and holistic approach and effective team work to minimize care suffering in the woman with PPCM. PPCM knowledge should be included in the healthcare professionals’ education curriculum, and all clinicians, especially midwives, should be equipped with the skills needed to identify PPCM for early referral to a specialist [11]. Further research on the heath care professional’s and especially the midwives’ perspective is recommended so that this condition will not be misdiagnosed or mismanaged. A follow-up examination to strengthen the results of the current study may solve the riddle of how subsequent care relationships and attitudes of healthcare professionals may be improved, for the benefit of affected women.

## Abbreviations

ECG: Electro-cardiogram
ECHO: Echocardiography
HF: Heart failure
ICCU: Intensive Coronary Care Unit
PCC: Person-centered care
PPCM: Peripartum cardiomyopathy
